# Supplementary material for: Mussel Shell-Derived Macroporous 3D Scaffold: Characterization and Optimization Study of a Bioceramic from the Circular Economy
Source: Mar Drugs. 2020 Jun 12;18(6):309. doi: 10.3390/md18060309 (PMC7344406; doi:10.3390/md18060309)
Supplement: Supplementary file 1 [file marinedrugs-18-00309-s001.pdf]

# SUPPORTING INFORMATION

# Mussel shell-derived macroporous 3D scaffold: characterization and optimization study of a bioceramic from circular economy

**Stefania Scialla [1,§], Francesca Carella [2], Massimiliano Dapporto [2], Simone Sprio [2], Andreana Piancastelli [2], Barbara Palazzo [3,‡], Alessio Adamiano [2], Lorenzo Degli Esposti [2], Michele Iafisco [2,*] and Clara Piccirillo [1,*]**

[1] Institute of Nanotechnology (NANOTEC), National Research Council (CNR), Campus Ecoteckne, Via Monteroni, Lecce, Italy; stefania.scialla@inl.int (S.S.)

[2] Institute of Science and Technology for Ceramics (ISTEC), National Research Council (CNR), Via Granarolo 64, 48018 Faenza, Italy; francesca.carella@istec.cnr.it (F.C.); massimiliano.dapporto@istec.cnr.it (M.D.); simone.sprio@istec.cnr.it (S.S.); andreana.piancastelli@istec.cnr.it (A.P.); alessio.adamiano@istec.cnr.it (A.A.); lorenzo.degliesposti@istec.cnr.it (L.D.E.)

[3] Ghimas SpA, C/O Ditech S.c.a.r.l., Campus Ecotekne, Lecce, Italy; barbara.palazzo@enea.it (B.P.)

* Correspondence: michele.iafisco@istec.cnr.it (M.I.); clara.piccirillo@nanotec.cnr.it (C.P.); Tel.: +39-0546-699730 (M.I.); +39-0832-319833 (C.P.)

[§] Current affiliation: Advanced (magnetic) Theranostic nanostructures Lab, International Iberian Nanotechnology Laboratory, Av. Mestre José Veiga s/n 4715-330 Braga, Portugal

[‡] Current affiliation: ENEA, Division for Sustainable Materials, Research Centre of Brindisi, 72100, Italy

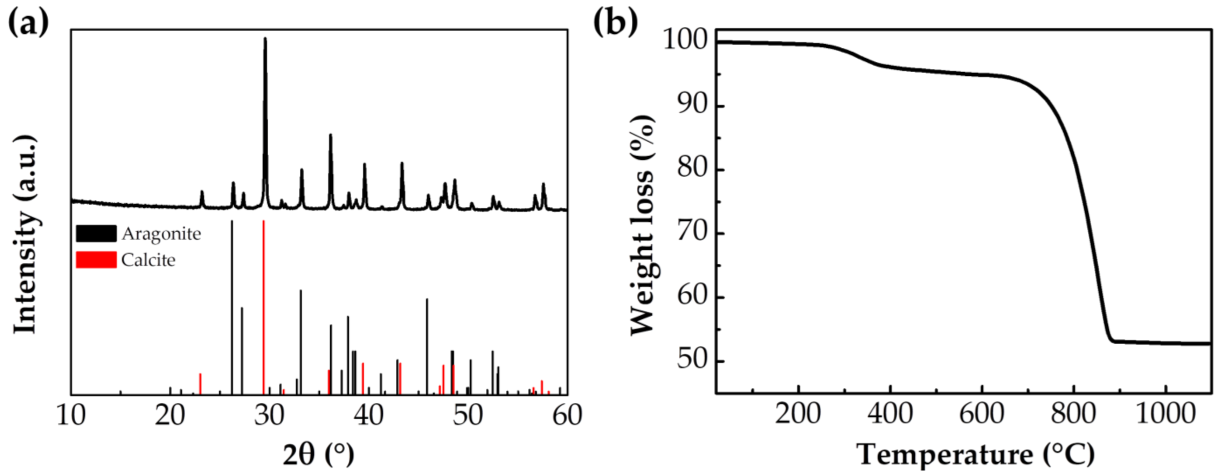


**Figure S1.** (a) XRD pattern and (b) TGA curve of raw mussel shells. In panel (a) the reference pattern of calcite (ASTM Card file No. 05-0586) and aragonite (ASTM Card file No. 041-1475) are also showed.

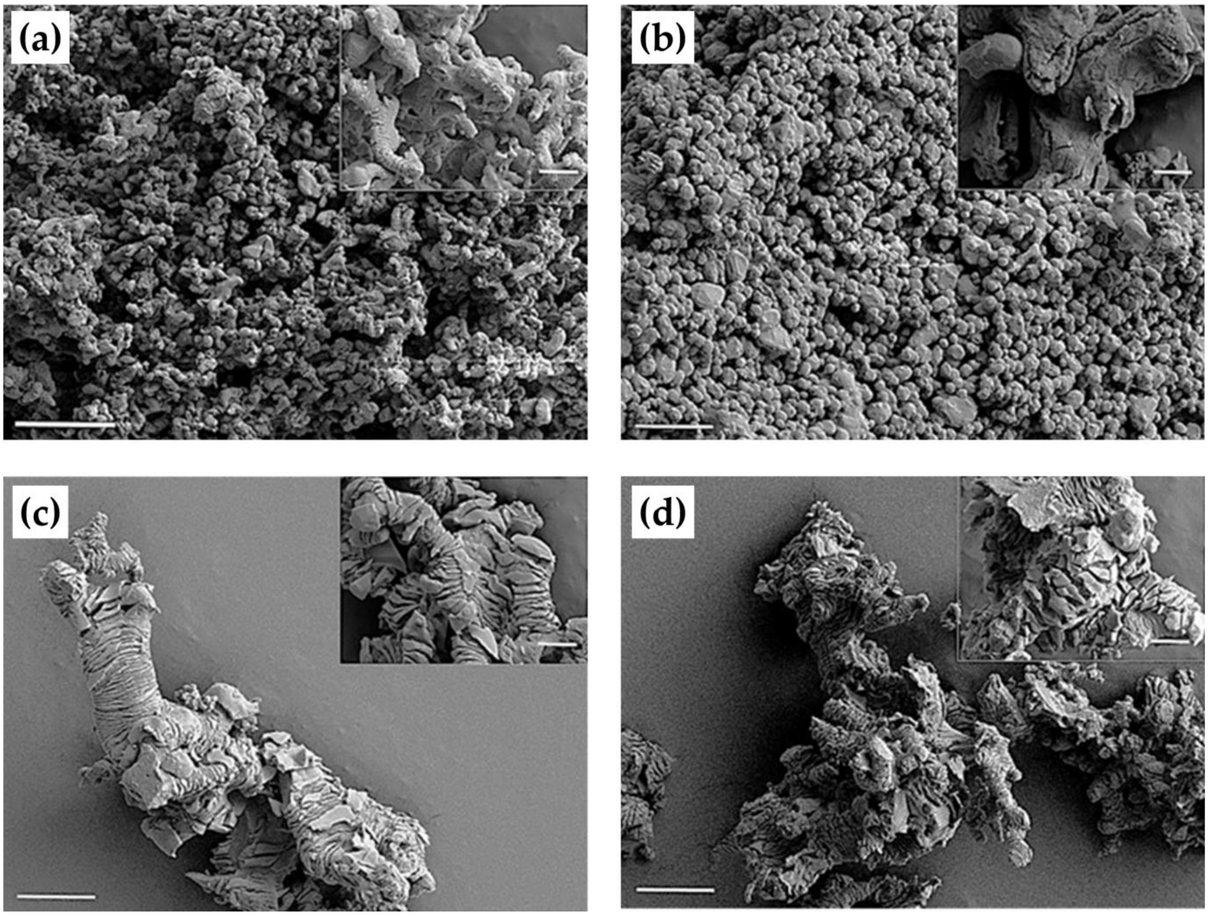


**Figure S2.** SEM micrographs of CaO samples prepared with different treatments, (a) S_A_700, (b) S_A_1000, (c) S_N_700, (d) S_N_1000 [main images: magnification 5kX, scale bar 10 µm; insert: magnification 25 kX, scale bar 1 µm].

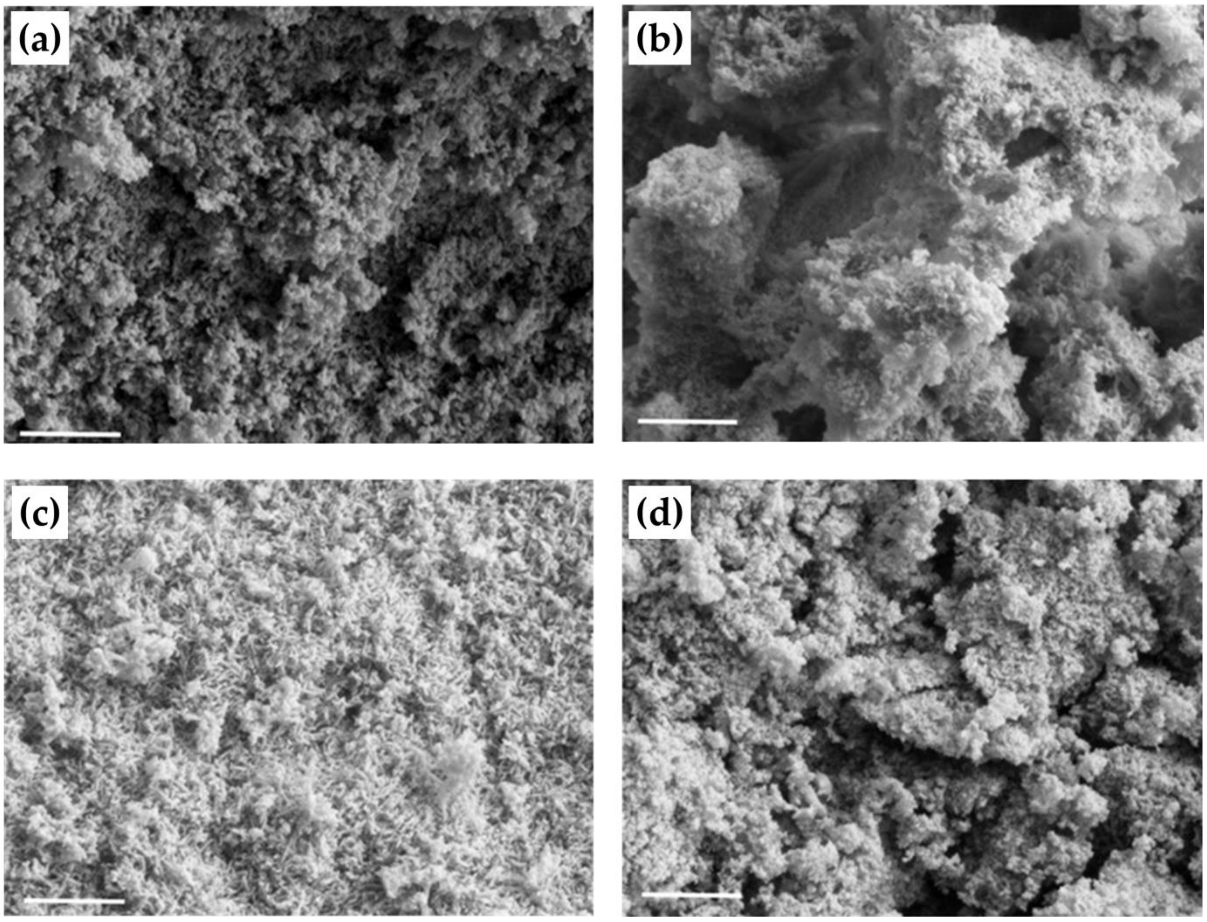


**Figure S3**. SEM micrographs of HA samples prepared with different protocols; (a) HA_1, (b) HA_2, (c) HA_3, (d) HA_4 [magnification 50 kX, scale bar 1 μm].

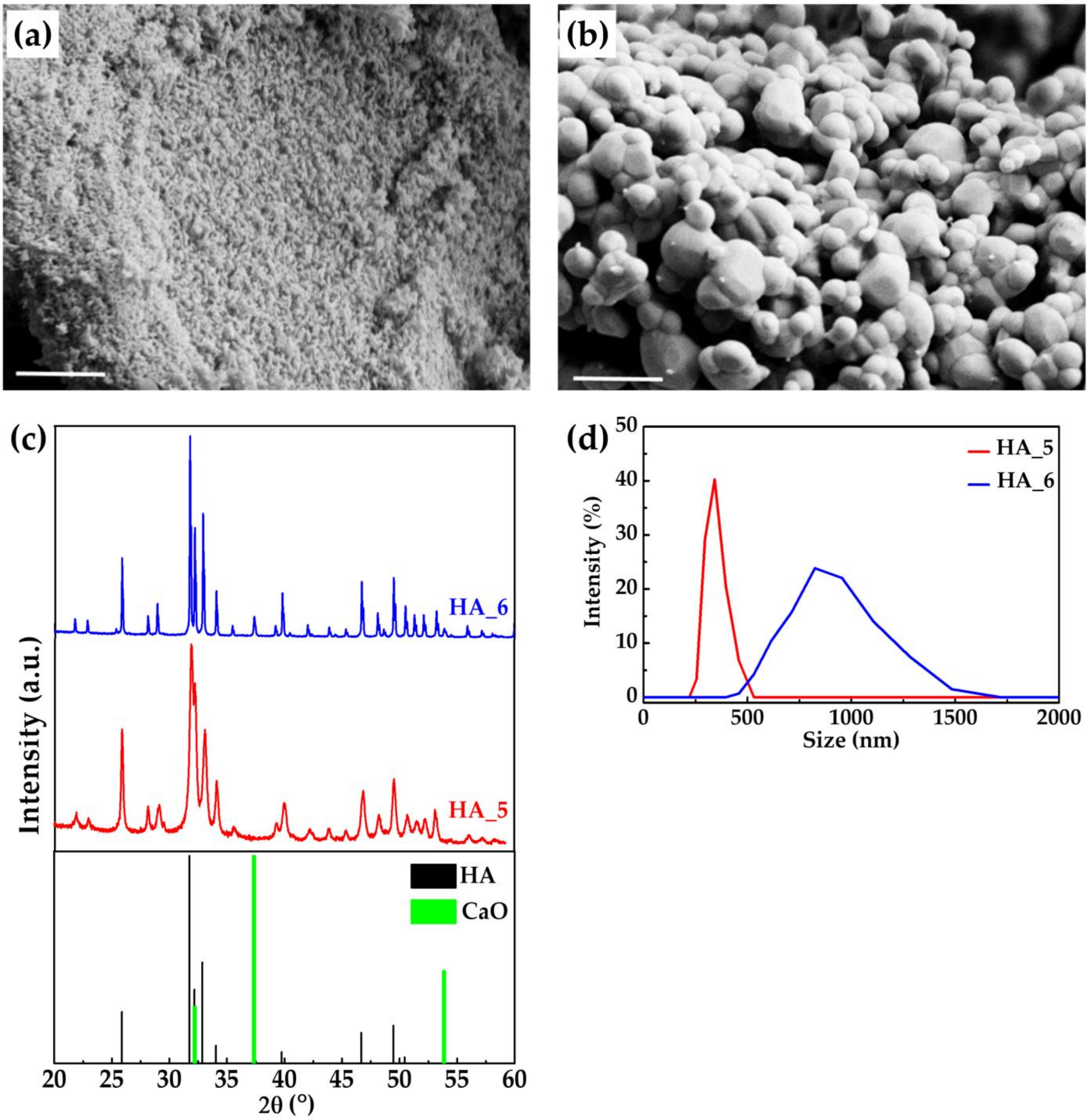


**Figure S4**. SEM micrographs of HA sample prepared at pilot scale (HA_5) (a) before and (b) after calcination at 1000 °C for 1 h (HA_6) [magnification 50 kX, scale bar 1 µm]; (c) XRD patterns of HA_5 and HA_6 samples, XRD pattern of standard of HA (ASTM Card file No. 09-0432) and CaO (ASTM Card file No. 37-1497) is also showed; (d) size distribution of HA_5 and HA_6 samples suspended in Dolapix 4 wt%.

**Table S1**. Chemical composition of the raw mussel shells and CaO samples

| Samples | Ca (wt%) | Mg (wt%) | Na (wt%) | P (wt%) | Se (wt%) | Sr (wt%) |
|---|---|---|---|---|---|---|
| Raw mussel shells | 34.62±0.74 | 0.11±0.01 | 0.55±0.04 | 0.46±0.04 | 0.16±0.21 | 0.09±0.01 |
| S_A_700 | 42.85±1.90 | 0.09±0.01 | 0.29±0.03 | 0.04±0.03 | 0.71±0.23 | 0.10±0.01 |
| S_A_1000 | 48.26±0.40 | 0.10±0.01 | 0.16±0.06 | 0.05±0.01 | 0.59±0.34 | 0.11±0.01 |
| S_N_700 | 40.70±3.73 | 0.14±0.01 | 0.39±0.10 | 0.17±0.06 | 0.49±0.15 | 0.11±0.01 |
| S_N_1000 | 43.98±0.86 | 0.12±0.01 | 0.20±0.03 | 0.02±0.01 | 0.37±0.22 | 0.11±0.01 |

List of elements analyzed and their relative LOD (Limit of detection of the element in the sample):
Al, As, Ba, B, Be, Ca, Cd, Co, Cu, Cr, Fe, Mg, Mn, Na, Ni, P, Pb, Se, Sr, V, Zn.

LOD was 1 ppm for As, B, Be, P, Pb, Se
LOD was 0.05 ppm for Al, Ba, Ca, Cd, Co, Cu, Cr, Fe, Mg, Mn, Na, Ni, Sr, V, Zn
